# Including the criminal justice‐involved at the HIV policy, research and service delivery table

**DOI:** 10.1002/jia2.25145

**Published:** 2018-06-07

**Authors:** Derrick T Ndlovu, Christopher J Hoffmann

**Affiliations:** ^1^ Zonk`izizwe Odds Development (ZOD) Johannesburg South Africa; ^2^ Department of Medicine Johns Hopkins University School of Medicine Baltimore MD USA

**Keywords:** HIV, HIV care continuum, key and vulnerable populations, people in prisons, community‐based participatory research, stigma

Representation in policy, research and service delivery from populations most affected by HIV is essential to optimize responses to the HIV pandemic. For years, research and policy has been strengthened through the advocacy of communities most affected by HIV 1. However, not all highly affected groups have been able to fully participate. One such highly affected key population is the criminal justice involved, defined as individuals who are currently detained or have been in detention. This group has had minimal representation on policy committees and limited visibility at major HIV conferences. Greater involvement of criminal justice involved people living with HIV around the world is essential to most effectively address the specific HIV treatment and prevention challenges faced by this population.

Globally, the criminal justice involved population is large and is disproportionately burdened with HIV. There are an estimated 10.3 million sentenced inmates; adding former inmates to this population increases it by as much as 10‐fold 2. The HIV prevalence in this population generally exceeds that of local non‐detained populations 3. For example, in the United States approximately 14% of all people living with HIV pass through the correctional system annually 4, 5. In southern Africa, a quarter or more of inmates are living with HIV; high prevalence has also been reported from countries in West and East Africa 4. In Eastern Europe and Central Asia approximately 5% to 19%, in the Asia Pacific Region 3% to 9% and in Latin America and the Caribbean 3% to 11% of inmates are living with HIV 4, 6. The reasons that justice involved individuals have a higher HIV prevalence than the general populations include living in impoverished and marginalized areas, lack of access to prevention, sexual violence (in and outside of correctional environments) and injection drug use 4, 7.

Challenges specific to justice involved populations during incarceration have global commonality as well as regional variation. In sub‐Saharan Africa overcrowding, poor nutrition and limited access to health services are common 8. Crowded conditions, lack of infection control and high HIV prevalence also create a high risk environment for TB transmission in Africa and similarly in Eastern Europe and Asia 6, 9, 10. Physical, psychological and sexual abuse by corrections staff and other inmates (sometimes officially or unofficially empowered by corrections staff) threaten the physical and mental health of inmates around the world 7, 11. Access to HIV prevention in the form of condoms, pre‐exposure prophylaxis, post‐exposure prophylaxis and sexual violence reduction programming are often inadequate or generally lacking in correctional facilities in most of the world. Punitive policies regarding sexuality and limitations on access to medical care further increase vulnerability to HIV infection and progression of HIV disease 7, 8. Furthermore, in settings with ART availability, transfers between pre‐trail detention and post‐sentencing detention or between correctional facilities after sentencing may lead to gaps in medication provision 12. This prevented ART adherence for one of the authors (DTN), when he was transferred from one correctional facility to another in South Africa and created an adherence challenge when he was eventually released from incarceration.

Vulnerability to HIV infection and poor health outcomes persist beyond confinement. Discriminatory hiring, health service, housing and social service practices as well as stigma related to justice involvement are routinely encountered following release, extending the potential health impact of incarceration long after release 2. In addition, incarceration is characterized by psychological adaptations including dependence on institutional structure with diminished self‐initiative, hypervigilance, interpersonal distrust, alienation, psychological distancing and social withdrawal 13, 14.

Translating a general awareness of the challenges faced by the justice involved into the development of optimal policy, research and programmes is best achieved through inclusion of this population in decision making. However, giving voice to this critical perspective will not come easily. Following incarceration, opportunities for involvement in policy, service delivery and research structures often remain out of reach due to the psychosocial impact of incarceration, stigmatization of being an ex‐inmate and the challenges of daily survival. These are challenges that one of the authors, TD Ndlovu has faced in advocating for the health of justice involved populations. Lack of familiarity with organizations that work in policy, research, or advocacy, and lack of skills typically desired by such organizations can further hamper meaningful and sustained engagement. To make the voices of the justice involved appropriately heard will require investment in dedicated efforts to nurture representation from this population and facilitate ongoing engagement. Such activities may include financial support for individuals and advocacy groups, travel stipends to conferences, training, guidance from policy and research organizations, and designated representation on policy panels that discuss the justice involved.

The phrase “nothing about us without us” has been used by key populations to highlight the importance of having the affected at the table. To move towards ending AIDS through a nothing about us without us approach, the justice involved population should be actively involved in policy making, programme planning, service design and research.

## Competing Interest

Both authors declare no conflicts of interest.

## Author's contributions

Both authors contributed equally to this work. DTN and CJH conceptualized the viewpoint. DTN and CJH contributed equally to the first draft and subsequent revisions. CJH performed the literature search. All authors have read and approved the final manuscript.
